# Predicting treatment Response based on Dual assessment of magnetic resonance Imaging kinetics and Circulating Tumor cells in patients with Head and Neck cancer (PREDICT-HN): matching ‘liquid biopsy’ and quantitative tumor modeling

**DOI:** 10.1186/s12885-018-4808-5

**Published:** 2018-09-19

**Authors:** Sweet Ping Ng, Houda Bahig, Jihong Wang, Carlos E. Cardenas, Anthony Lucci, Carolyn S. Hall, Salyna Meas, Vanessa N. Sarli, Ying Yuan, Diana L. Urbauer, Yao Ding, Shane Ikner, Vi Dinh, Baher A. Elgohari, Jason M. Johnson, Heath D. Skinner, G. Brandon Gunn, Adam S. Garden, Jack Phan, David I. Rosenthal, William H. Morrison, Steven J. Frank, Katherine A. Hutcheson, Abdallah S. R. Mohamed, Stephen Y. Lai, Renata Ferrarotto, Michael P. MacManus, Clifton D. Fuller

**Affiliations:** 1 Department of Radiation Oncology, University of Texas MD Anderson Cancer Center, 1515 Holcombe Blvd, Houston, TX 77030 USA; 2 Department of Radiation Physics, University of Texas MD Anderson Cancer Center, Houston, TX USA; 3 Department of Breast Surgical Oncology, University of Texas MD Anderson Cancer Center, Houston, TX USA; 4 Department of Biostatistics, University of Texas MD Anderson Cancer Center, Houston, TX USA; 5 Department of Diagnostic Radiology, University of Texas MD Anderson Cancer Center, Houston, TX USA; 6 Department of Head and Neck Surgery, University of Texas MD Anderson Cancer Center, Houston, TX USA; 7 Department of Thoracic Head and Neck Medical Oncology, University of Texas MD Anderson Cancer Center, Houston, TX USA; 8 Department of Radiation Oncology, Peter MacCallum Cancer Centre, Melbourne, Australia

**Keywords:** Magnetic resonance imaging, Circulating tumor cells, Head and neck cancer, Biomarker

## Abstract

**Background:**

Magnetic resonance imaging (MRI) has improved capacity to visualize tumor and soft tissue involvement in head and neck cancers. Using advanced MRI, we can interrogate cell density using diffusion weighted imaging, a quantitative imaging that can be used during radiotherapy, when diffuse inflammatory reaction precludes PET imaging, and can assist with target delineation as well. Correlation of circulating tumor cells (CTCs) measurements with 3D quantitative tumor characterization could potentially allow selective, patient-specific response-adapted escalation or de-escalation of local therapy, and improve the therapeutic ratio, curing the greatest number of patients with the least toxicity.

**Methods:**

The proposed study is designed as a prospective observational study and will collect pretreatment CT, MRI and PET/CT images, weekly serial MR imaging during RT and post treatment CT, MRI and PET/CT images. In addition, blood sample will be collected for biomarker analysis at those time intervals. CTC assessments will be performed on the CellSave tube using the FDA-approved CellSearch® Circulating Tumor Cell Kit (Janssen Diagnostics), and plasma from the EDTA blood samples will be collected, labeled with a de-identifying number, and stored at − 80 °C for future analyses.

**Discussion:**

The primary objective of the study is to evaluate the prognostic value and correlation of weekly tumor response kinetics (gross tumor volume and MR signal changes) and circulating tumor cells of mucosal head and neck cancers during radiation therapy using MRI in predicting treatment response and clinical outcomes. This study will provide landmark information as to the utility of CTCs (‘liquid biopsy) and tumor-specific functional quantitative imaging changes during treatment to guide personalization of treatment for future patients. Combining the biological information from CTCs and the structural information from MRI may provide more information than either modality alone. In addition, this study could potentially allow us to determine the optimal time to obtain MR imaging and/ or CTCs during radiotherapy to assess tumor response and provide guidance for patient selection and stratification for future dose escalation or de-escalation strategies.

**Trial registration:**

Clinicaltrials.gov (NCT03491176). Date of registration: 9^th^ April 2018. (retrospectively registered). Date of enrolment of the first participant: 30^th^ May 2017.

## Background

Head and neck squamous cell carcinoma (HNSCC) has an estimated incidence of approximately 50,000 annual cases in the United States, with an annual mortality estimated at 11,400 persons [1]. Recently, data has emerged that human papilloma virus (HPV) associated head and neck cancers [2], while demographically on the rise, are comparably more radiocurable than traditional non-HPV-associated HNSCC, with observed 3-year survival in Phase III cooperative group secondary analyses of 82% in patients treated with radiotherapy, compared to 57% in HPV- patients treated equivalently [3].

Currently there are numerous phase II/III studies for HPV-positive oropharyngeal cancers evaluating the possibility of de-intensification and/or radiation dose reduction in this subgroup of tumors with better prognosis than their non-HPV associated counterparts. Conversely, there are not many current studies or advances in the treatment of non-HPV associated head and neck cancers. With advances in imaging and radiation treatment planning and delivery, adaptive radiation planning may allow for dose escalation in this group of patients with more radioresistant tumors.

### Magnetic resonance imaging (MRI)

Magnetic resonance imaging (MRI) has improved capacity to visualize tumor and soft tissue involvement in head and neck cancers [4]. Using advanced MRI, we can interrogate cell density using diffusion weighted imaging, a quantitative imaging that can be used during radiotherapy, when diffuse inflammatory reaction precludes PET imaging, and can assist with target delineation as well. Correlation of circulating tumor cells (CTCs) measurements with 3D quantitative tumor characterization could potentially allow selective, patient-specific response-adapted escalation or de-escalation of local therapy, and improve the therapeutic ratio, curing the greatest number of patients with the least toxicity. Our preliminary findings showed that functional MRI using intravoxel incoherent motion (IVIM) analysis can indicate early-therapy complete response amount patients with HPV-positive oropharyngeal cancer, allowing identification of those likely to derive benefit from treatment modification [3]. However, this has not been validated in the non-HPV related head and neck cancer. As part of a systematic effort to develop MRI applications in head and neck cancers, we have implemented several protocols using pre- and mid-therapy MRI for patients undergoing radiotherapy. In our dataset (consistent with data from a previous adaptive radiotherapy cohort), some patients complete resolution of clinical and radiographically evident disease on mid-therapy (week 3–4) imaging, with the vast majority of observed patients exhibiting interval tumor shrinkage [5].

### Nutritional and hematologic markers

Active cancer treatment was found to directly affect various laboratory markers, including acute phase reactant (c-reactive protein (CRP), erythrocyte sedimentation rate (ESR)), albumin, and neutrophil, lymphocyte, monocyte and platelet counts. These changes are not just a direct effect of treatment, but were found to have prognostic value in head and neck cancer patients. Individual studies showed that higher pre-treatment neutrophil-to-lymphocyte ratio (NLR) and platelet-to-lymphocyte ratio (PLR) were associated with a poorer clinical outcome [6–9]. Our retrospective study has shown that elevated pre-treatment platelet count is associated with poorer locoregional control and distant free metastasis outcomes, compared to non-elevated platelet count [10].

There have been studies, majority retrospective, correlating patients’ clinical outcomes with pre-treatment and post-treatment individual blood markers [6–12]. However, there has been no study correlating these blood results as a whole with patients’ radiological tumor response during treatment and clinical outcomes. With the availability of serial MRI, we are able to track and directly correlate the potential changes in laboratory markers with radiologic tumor response. We aim to assess the prognostic value of these laboratory markers.

### Circulating tumor cells (CTCs) as liquid biopsy

Cancer metastasizes when tumor cells are shed from the primary tumor, enters the circulation, and established a new site of growth [13]. CTCs are detectable in many cancers, including head and neck cancers [14–16], where they can have prognostic significance. CTCs may be present at diagnosis [17, 18] or may have been mobilised into the circulation during treatment, as evidenced by a study in lung cancer by Martin et al. [19]. In breast [20–22], prostate [23, 24] and colorectal cancers [25], CTC was validated as a prognostic factor.

A review of CTC studies in head and neck cancers by Wu et al. [26] have shown that the recurrence/ metastasis rate in CTC-positive patients was significantly higher than CTC-negative patients. In addition, the presence of CTC suggested a worse disease-free survival for head and neck cancer patients [16, 17, 26]. However, majority of these studies and reviews in head and neck cancer were retrospective analysis. Furthermore, p16 status was not reported in these studies and patients had a variable radiotherapy course and/or systemic agents. To date, there has been no prospective study of the significance of CTCs and its correlation with radiological response and clinical outcomes. In this study, we aim to assess the presence of CTC pre-treatment, during and post-treatment in this study and to correlate CTCs with radiological and clinical outcomes.

With the availability of the MRI simulation machine at the MD Anderson Cancer Centre, we will be able to collect weekly MR images to assess tumor size and signal kinetics in this group of patients. Normal tissue signal kinetics will also be assessed and correlated to treatment toxicity. Additionally, blood specimens will be collected to assess, quantify and validate the possible blood biomarkers that correlates with patients’ tumor kinetics and clinical outcomes. This prospective study will allow us to collect both imaging and blood biomarkers (‘liquid biopsy’) information simultaneously, permitting accurate assessment and correlation of these markers in time.

## Methods/ design

### Study design and consent

**Fig. 1:**
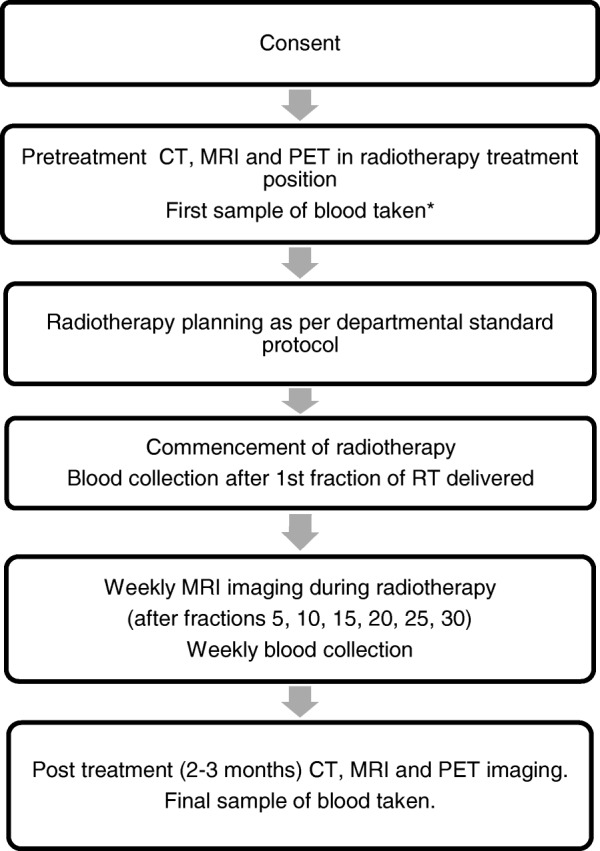
Research protocol workflow. * If receiving induction chemotherapy, first sample of blood collected pre-chemotherapy

The proposed study is designed as a prospective observational study and will collect pretreatment CT, MRI and PET/CT images, weekly serial MR imaging during RT and post treatment CT, MRI and PET/CT images (Fig. 1). In addition, blood sample will be collected for biomarker analysis at those time intervals. There will be no change to patients’ standard of care treatment plan in this study. Patients will be treated in standard departmental protocol.

### Hypotheses

1. Changes in CTC counts and CTC clusters during radiotherapy will have prognostic significance.
2. Changes in tumor signals detected on serial MRI scans during radiotherapy will be predictive of outcomes in head and neck cancer.
3. A relationship can be detected between serial CTC assays and changes in tumor characteristics detected on sequential MRI during radiotherapy.

### Study objectives

#### Primary objective

To assess the prognostic value and correlation of weekly tumor response kinetics (gross tumor volume and MR signal changes) and circulating tumor cells of mucosal head and neck cancers during radiation therapy using MRI in predicting treatment response and clinical outcomes.

#### Secondary objectives

- To assess MR imaging kinetics (tumor volume and signal changes) as a marker of tumor locoregional control
- To correlate the presence and amount of CTCs with radiological tumor kinetics during treatment and subsequent treatment outcomes
- To determine the optimal time to obtain MR imaging and/ or CTCs during radiotherapy to assess tumor response
- To correlate normal tissue MR signal changes during treatment with acute and late toxicity of treatment
- To explore tumor and normal tissue radiomics in pre-treatment and during treatment MR images as a tool to predict for subsequent treatment outcomes and toxicity
- To assess the consistency of MR signal information obtained during radiotherapy and the possible dose distribution changes over time, as a results of tumor volume and body habitus change, in a cohort of head and neck patients

### Subject selection

#### Inclusion criteria

- Biopsy proven diagnosis of squamous cell carcinoma (SCC) of head and neck mucosa or unknown primary of the head and neck (suspected mucosal primary). Clinical evidence should be documented, and may consist of imaging, endoscopic evaluation, palpation, and should be sufficient to estimate the size of the primary for purposes of T staging.
- Age ≥ 18 years
- No distant metastases, based on routine staging workup.
- Consent for blood collection for biomarker analysis
- No head and neck surgery of the primary tumor or lymph nodes except for incisional or excisional biopsies
- Eastern Cooperative Oncology Group (ECOG) performance status of 0–2.
- Consented to curative intent radiotherapy
- For females of child-bearing age, a negative pregnancy test

#### Exclusion criteria

- Previous radiation treatment for head and neck mucosal primary cancers within the past 5 years
- Pregnant or breast-feeding females
- Contraindications to MR imaging (e.g. implanted metallic prostheses, pacemakers, defibrillators, or stimulators)
- Significant claustrophobia

#### Criteria for removal from the study

Patients will be removed from the study if they withdraw consent or if radiation treatment is discontinued prior to completion of the study.

### Image acquisition

Patients will have standard of care pretreatment and staging workup imaging including CT, MRI and PET/CT. During RT, patients will be treated using standard of care dose fractionation as determined by pathology and stage. Weekly MRI imaging will be obtained during treatment. Weekly MRI will be acquired for each patient in treatment position using the MAGNETOM Aera 1.5 T MR scanner (Siemens Healthcare, Erlangen, Germany) with two 4-channel large flex phased-array coils and 32-channel phase-array spine coil (Table 1).

**Table 1:** MRI sequences to be obtained during the study

Parameter	T1	T2	DWI (BLADE)
Slice Orientation	Axial	Axial	Axial
Field of View (mm)	256	256	256
Voxel Size (mm)	1 × 1 × 2	1 × 1 × 2	2 × 2 × 4
Recon Voxel Size (mm)	0.5 × 0.5 × 1	0.5 × 0.5 × 2	2 × 2 × 4
Parallel Imaging	No	Yes; Factor 2	No
Slice number	240	120	25
Fold-over Direction	AP	AP	N/A
Slice Oversampling	100%	No	No
Shim	Auto	Auto	Auto
Scan Mode	3D	M2D	M2D
Technique	GRE	SE	BLADE
Fast Imaging Mode	No	TSE	TSE and EPI
Echoes	1	1	1
Flip Angle (deg)	20	90	90
TR (ms)	7.38	4800	5400
Echo Time (ms)	4.77	80	50
Fat Suppression	No	No	Yes
b-values (s/mm^2^)	N/A	N/A	0, 800
NEX	1	1	8
Geometry Correction	3D	2D	No
Echo Train Length	1	15	15
Percent Sampling (%)	80	90	100
Pixel Bandwidth (Hz)	400	300	1220
Scan Duration (min)	6:05	4:48	7:08

As per standard institutional clinical follow up, imaging (CT, MRI and/or PET) will be obtained at 2–3 months after completion of radiation therapy.

### Blood samples collection and analysis

**Fig. 2:**
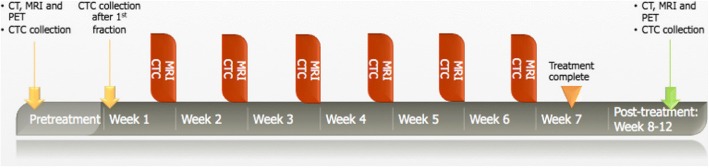
Research schema

Whole blood will be collected aseptically by venipuncture or from a venous port. Standard of care monitoring of routine blood parameters will be performed weekly. Blood will be collected at several time points: routine pre-treatment visit, after 1st fraction of radiotherapy, weekly during radiotherapy and at routine post-treatment visit (Fig. 2). An additional vial of 10 ml CellSave preservation tube (for CellSearch® CTC detection) and two 10 ml EDTA tubes will be collected. CTC assessments will be performed on the CellSave tube blood, and plasma from the EDTA blood samples will be collected, labeled with a de-identifying number, and stored at − 80 °C for future analyses. Research samples may include any or all of the designated samples. Any or all samples collected will result in a patient being eligible. A missed sample will not change eligibility status.

Wherever possible, blood samples will be collected at the same time as routine blood draws and/ or during intravenous cannula insertion procedure, thus minimizing patient stress and eliminating an additional invasive test for the patient. However, if a blood draw is not planned, one will be scheduled for this study. Nutritional and other hematologic blood marker parameters will be collected as part of routine blood draws.

10 ml of whole blood collected in the CellSave (Menarini-Silicon Biosystems) preservation tube will be analyzed using the FDA-approved CellSearch® Circulating Tumor Cell Kit (Menarini-Silicon Biosystems).

### Evaluation during study

Table 2 summarizes the pre-treatment (initial screening, baseline measures prior to enrollment), on treatment, post-treatment phases of the trial, and the evaluations that would be required during these phases.

**Table 2:** Summary of pre-, during and post evaluations during the study

	Baseline	Weekly treatment visits	Follow Up (2–3 months)
Physical examination with or without nasoendoscopy	X	X	X
Weight and BMI	X	X	X
Complete blood count with differential			
- Hemoglobin			
- Neutrophil count			
- Platelet count			
- Monocyte count	X	X	X
Basic Metabolic Panel			
- Electrolytes			
- Liver function tests			
- Albumin			
- ESR			
- CRP	X	X	X
Blood biomarker collection:			
- 10 ml CellSave tube			
- 2 × 10 ml EDTA tubes	X	X	X
Pregnancy test (female of childbearing age)	X		
Contrast CT head and neck	X		X
18F-FDG PET/CT *(optional)*	X		X
MRI of the head and neck	X	X	X

Treatment-related symptom scores and quality of life will be assessed weekly during treatment and at each routine follow up after completion of therapy. Clinical examination and radiological findings indicative of local, locoregional, and/or distant metastatic disease will be recorded.

### Statistical considerations

The primary objectives of this study are to assess the prognostic value and correlation of weekly tumor kinetics (TK) on imaging, and blood biomarkers of head and neck cancers during RT with treatment response and clinical outcomes. Tumor kinetics is the weekly decrease or increase in tumor volume and MR signal after initiation of radiotherapy, again evaluated using MRI. The blood biomarkers of interest are CTCs.

This study will enroll 100 patients. Given the expected toxicity of treatment and patients’ commitment to weekly imaging, it is estimated that 50–60% of enrolled patients may not complete the study. The study will close when we have 40 patients who completed the study. It is anticipated that enrolment will require 24 months to complete. Assuming only 40% of the subjects will complete the study and that 80% of subjects will have complete response, we will have 80% power using a 2-sample t-test with *α* = 0.1 to detect a 1 standard deviation difference between response cohorts with regard to weekly tumor kinetics and blood biomarkers.

We will use graphs and descriptive statistics to evaluate the relationship between TK and CTCs with response. It is expected that 80% of patients will have complete response (CR) to treatment, so the initial analysis will compare those with CR to all others. If we have a sufficient number of patients to separate stable disease (SD) and progressive disease (PD) from partial response (PR), we will conduct analyses separating patients with PR from those with SD/PD. Ninety percent confidence intervals (CIs) will be calculated for mean differences of TK and CTC counts between response cohorts at the time point. Logistic model will be fitted to examine the predictivity of TK and CTCs on response, while adjusting for potential confounders if the data permit. Prior to analysis, data will be transformed as needed.

We will also use generalized linear mixed models (GLMMs) to evaluate TK and CTCs over time and to assess whether a difference exists between patients with CR and those without CR. The primary analysis will use LMMs to assess whether differences exist by response cohort, although if the data permit, GLMMs will also be used to describe the ability of TK and CTCs to predict response. Additionally, if the data do not show a linear trend of TK or CTCs over time, curvilinear models or models using splines will be examined to obtain the best fit for the data.

Secondary objectives include assessing the relationship of TK and CTCs with locoregional control and correlating CTCs with TK during treatment. Locoregional control is defined as 2-year disease-free survival (DFS), and DFS is measured from date of first treatment to date of death, disease progression or recurrence, whichever occurs first. If a patient does not experience progression, recurrence or death, that patient will be censored in the analysis and disease-free survival is measured from date of first treatment until last date of known patient status. Joint longitudinal and survival models will be created to examine the trajectory and intercept of TK and CTCs during radiation therapy with DFS. Longitudinal modeling using a log link function will be used to assess the relationship between TK and the number of CTCs. In this model, weekly number of CTCs will be the outcome and both TK and time will be a predictor variable. The data will be analyzed using Tobit models if we find CTC count is zero-inflated.

### Data confidentiality

Strict patient confidentiality will be maintained. Patient confidentiality will be respected at all times. Patient’s name and medical record number will be removed from any stored data. Cases will coded by anonymous study number using a key kept separate from the database. Data will be stored on a password and firewall protected computer. Only the collaborators and the research team of this study will have access to patient information in order to extract the data from the medical records, and only information relevant to this protocol will be examined. All protected health information will be de-identified prior to releasing outcomes of the study. MRI data will be directly stored on an MD Anderson encrypted hard drive (purchased for the study). There will be no paper records of data with personal identifiers. The data will be stored for up to 5 years after the end of the study. Until then, the database will be password protected on a limited access computer.

## Discussion

Recent findings have shown that HNSCC associated with HPV, although demographically on the rise, can be more readily cured with radiation than the non-HPV- associated counterpart [3]. Currently phase II/III studies are ongoing for HPV-related oropharyngeal cancers to evaluate the possibility of treatment de-intensification or radiation dose reduction for such patients. However, fewer advances are being sought for HPV-non-associated HNSCC. Advances in imaging and radiation planning and delivery, with adaptive radiation planning, may allow safe dose escalation for such patients. Currently, no validated method has been found to identify patients at risk of incomplete tumor response during treatment, thereby representing a significant unmet need.

The ability of MRI to detect and visualize inter-fraction treatment response and soft tissues with high resolution, in combination with previously established methods for patient immobilization at the level required for precision radiotherapy, represents an advantage over the current standard of non-contrast CT-based radiotherapy. The availability of the MR simulator at MD Anderson Cancer Center provides an unprecedented opportunity to practically and readily acquire high- quality and high-frequency MR images while patients are in the treatment position, using novel imaging/treatment instruments. Cell density can be assessed via diffusion weighted imaging, when diffuse inflammation during treatment precludes PET imaging, and can assist with target delineation as well. Correlation of CTC measurements with 3D quantitative tumor characterization could potentially allow selective, patient-specific response-adapted escalation or de-escalation of local therapy, and improve the therapeutic ratio, curing the greatest number of patients with the least toxicity.

Cancer metastasis involves tumor cells being shed from the primary tumor, entering the circulation, and establishing new sites of growth. CTCs can be detected in head and neck cancer [14–16] and can have prognostic significance. CTCs may be present at diagnosis [17, 18] or may have been mobilized into the circulation during treatment [19, 27]. Wu et al. [26] have shown that the recurrence/ metastatic rate in CTC-positive patients was significantly higher than CTC-negative patients with head and neck cancers. In addition, the presence of CTC suggested a worse disease-free survival for HNSCC patients [16, 17, 26]. However, majority of these studies and reviews in head and neck cancer were in the setting of either pre- or post-definitive treatment and there has been no consistency with regards to the optimal timing of CTC testing for prognostic stratification. Simulated case scenarios have also shown no consensus with regards to timing and frequency of CTC testing and the infrequent monitoring of CTCs in the current literature may miss clinically relevant fluctuations in CTC counts [28]. To date, no prospective studies have been performed to identify the optimal frequency for CTC testing and to correlate CTC results with serial radiological response and clinical outcomes in patients with head and neck cancer during definitive radiotherapy. A systemic biomarker (‘liquid biopsy’) that could identify patients who would respond (or not) to local therapy (e.g., serum CTCs as a surrogate of locoregional control) would be actionable for assessing response during and after treatment, and permit response-adapted therapy.

The significance and innovation of the proposed research is that it is the first to correlate on- treatment serial imaging with serial CTC measurements in patients with potentially curable non-metastatic HNSCC. If completed successfully, this study will provide landmark information as to the utility of CTCs (‘liquid biopsy) and tumor-specific functional quantitative imaging changes during treatment to guide personalization of treatment for future patients. Combining the biological information from CTCs and the structural information from MRI may provide more information than either modality alone.

## Abbreviations

BMI: Body mass index
CR: Complete response
CRP: C-reactive protein
CT: Computed tomography
CTC: Circulating tumor cell
DFS: Disease free survival
DWI: Diffusion-weighted imaging
ESR: Erythrocyte sedimentation rate
FDG: Fluorodeoxyglucose
GLMM: Generalized linear mixed model
HNSCC: Head and neck squamous cell carcinoma
HPV: Human papillomavirus
MRI: Magnetic resonance imaging
NLR: Neutrophil-lymphocyte ratio
PD: Progressive disease
PET: Positron emission tomography
PLR: Platelet-lymphocyte ratio
PR: Partial response
SD: Stable disease
TK: Tumor kinetics
TR: Relaxation time
